# Editorial: Empowering individuals: promoting health literacy through curriculum and science communication

**DOI:** 10.3389/fpubh.2025.1632938

**Published:** 2025-06-13

**Authors:** Muriel Grenon, Padraig Murphy, Doras Sibanda, Aimee Pugh-Bernard

**Affiliations:** ^1^College of Science and Engineering, University of Galway, Galway, Ireland; ^2^School of Communications, Dublin City University, Dublin, Ireland; ^3^School of Education, University of KwaZulu-Natal, Durban, South Africa; ^4^Department of Immunology and Microbiology, University of Colorado Anschutz Medical Campus, Aurora, CO, United States

**Keywords:** science, communication, health, literacy, education, science communication

## 1 Introduction

Empowering individuals in society to make evidence-based decisions about their health and wellbeing is vital, particularly in the current context of an overwhelming amount of misinformation, disinformation, and conspiracy theories easily accessible on the internet and social media platforms (1). Effective science communication plays a key role in providing accurate information that empowers individuals to make informed decisions in everyday lives (2). The incorporation of science communication training within higher education for scientists and healthcare professionals directly impacts public trust in scientific and healthcare institutions, promoting informed decision-making and enhancing public health outcomes.

This Research Topic aimed to call attention to the educational approaches and real-life applications of science communication for scientists and health professionals, and their impact on society often through formal, non-formal, or informal science education initiatives. The implementation of curricular or extracurricular educational initiatives that promote teaching strategies for effective science communication aimed at equipping future professionals with the skills to translate complex scientific information into language that is accessible to the general public. Further, communication training that focuses on deeper forms of engagement helps scientists to understand and facilitate the social contexts to communication increasing inclusivity and reducing inequalities (3, 4). Acquiring these skills prepares scientists and health care professionals to serve as credible sources of information and as advocates for public health and wellbeing.

## 2 Contributions to the Research Topic

This Research Topic contains five peer-reviewed articles exploring the promotion of health literacy through different avenues. Covering different health specialties and international contexts, the Research Topic includes two original research papers, one study protocol, one brief research paper, and one perspective article and gives a diverse landscape on how different communications methods could achieve individual health empowerment.

- **Blended public health empowerment programme:** Alsouri et al. present a study that establishes the potential of blended programmes as impactful tools to improve professional skills in local community communication. They provide a detailed evaluation of the *Public Health Empowerment Program – Basic Field Epidemiology (PHEP-BFE)*, a blended learning initiative used within the *Eastern Mediterranean Public Health Network (EMPHNET)* in Iraq, Egypt, and Lebanon.
- **The public health impact of TV medical dramas:** Zago et al. studied popular medical dramas as they can shape how both the public and healthcare professionals think about health issues. While they often bring attention to important topics, they tend to focus more on dramatic emergencies than routine everyday health care or prevention. They identified exaggerated or misrepresented areas of real-life medicine that should be addressed to improve general public health literacy, including real causes of hospitalization, prevention and health promotion.
- **Improving trust and diversifying participation in clinical trials:** Gutnick et al. propose a study protocol based on community participation to improve a minority representation in clinical trials to promote social justice and equity. Their *Bridging Research Accurate information and Dialogue (BRAID)* model will be further developed in collaboration with underrepresented communities in New York, USA. The two-phase model engages the community in creating health messages by bringing their concerns to scientists and researchers. These messages will then be disseminated through the community by community based trusted messengers.
- **Designing better communication tools for health:** Pellegrini and Lovati outlines a plan for the design of a stakeholder engagement model (Health Social Laboratories), to account for the complexity of communication in the context of neurodegenerative disease as part of the *Hereditary* project in Europe. This study stresses the importance of including diverse voices, such as researchers, patients, caregivers, health institutions, policy experts, local government representatives, and underrepresented groups to identify challenges and work together to improve healthcare communication.
- **The critical importance of communication competency in epidemiology:** Abraham and Hlaing emphasize the repeated calls for epidemiology education to prioritize communication training, and make a case for essential skills to be fully embedded in epidemiology curricula. They list the key communication competencies that are crucial for future professionals to combat misinformation and promote public health. For example, the ability to convey scientific information in clear, accessible language to diverse public audiences should be taught instead of the current focus on research-oriented communication. In addition, teaching formats involving community partners should be introduced to provide students with practical, hands-on experience in public engagement and health communication.

## 3 Implications for future research and practice

While challenges exist to add more content into science and healthcare educational curriculum, it is an essential task to ensure the effective training of the next generation of scientists. The submissions here offer interesting insights into various methods and approaches that can be applied across different fields. There is strong representation of community involvement, as well as the role of traditional media. However, within this Research Topic, there is a noticeable lack of articles on science communication in the context of evolving media types and the implementation of science communication into educational curricula for scientists and medical professionals. While a wide variety of communication courses and programs exist for individuals focused on careers in communication, there is a lack of formal educational curricula in science communication for science and healthcare trainees and professionals. As an example, Abraham and Hlaing is the only article within this Research Topic that called attention to the need for science communication skills to be developed for students training in science and healthcare. This highlights the significant gap in science communication within the formal educational curriculum at universities, particularly for students pursuing science and healthcare professions (5, 6).

As public trust in science continues to decline, universities are beginning to understand the need to incorporate curricular or extracurricular science communication training into scientific and medical education, from beginning student to experienced faculty. Relying solely on trained communicators without scientific training is no longer sufficient. Effective science communication is a core competency for all scientists and healthcare professionals. Embedding science communication curriculum or workshop opportunities within educational programs will ensure that these skills are consistently developed from the start of their training.

## References

[1] Jabkowski P Domaradzki J Baranowski M. Religiosity and beliefs in medical conspiracy theories in 37 European countries during the COVID-19 pandemic. Humanit Soc Sci Commun. (2025) 12:434. 10.1057/s41599-025-04781-4

[2] Nutbeam D Lloyd JE. Understanding and responding to health literacy as a social determinant of health. Annu Rev Public Health. (2021) 42:159–73. 10.1146/annurev-publhealth-090419-102529 33035427

[3] Canfield KN Menezes S Matsuda SB Moore A Mosley AAN Dewsbury BM. Science communication demands a critical approach that centers inclusion, equity, and intersectionality. Front Commun. (2020) 5:2. 10.3389/fcomm.2020.00002

[4] Fähnrich B Wilkinson C Weitkamp E Heintz L Ridgway A Milani E. Rethinking science communication education and training: towards a competence model for science communication. Front Commun. (2021) 6:795198. 10.3389/fcomm.2021.795198

[5] Bankston A McDowell GS. Changing the Culture of Science Communication Training for Junior Scientists. J Microbiol Biol Educ. (2018) 19:43. 10.1128/jmbe.v19i1.1413 29904538 PMC5969424

[6] Kington RS Arnesen S Chou WS Curry SJ Lazer D Villarruel AM. Identifying credible sources of health information in social media: principles and attributes. NAM Perspectives Discussion Paper, National Academy of Medicine, Washington, DC (2021). 10.31478/202107a 34611600 PMC8486420

